# Wt1 Positive dB4 Neurons in the Hindbrain Are Crucial for Respiration

**DOI:** 10.3389/fnins.2020.529487

**Published:** 2020-11-30

**Authors:** Danny Schnerwitzki, Christian Hayn, Birgit Perner, Christoph Englert

**Affiliations:** ^1^Molecular Genetics Laboratory, Leibniz Institute on Aging–Fritz Lipmann Institute (FLI), Jena, Germany; ^2^Core Facility Imaging, Leibniz Institute on Aging-Fritz Lipmann Institute (FLI), Jena, Germany; ^3^Institute of Biochemistry and Biophysics, Friedrich-Schiller-University Jena, Jena, Germany

**Keywords:** Wilms tumor protein, Wt1, respiration, dB4 neurons, development

## Abstract

Central pattern generator (CPG) networks coordinate the generation of rhythmic activity such as locomotion and respiration. Their development is driven by various transcription factors, one of which is the Wilms tumor protein (Wt1). It is present in dI6 neurons of the mouse spinal cord, and involved in the coordination of locomotion. Here we report about the presence of Wt1 in neurons of the caudoventral medulla oblongata and their impact on respiration. By employing immunohistofluorescence staining, we were able to characterize these Wt1 positive (+) cells as dB4 neurons. The temporal occurrence of Wt1 suggests a role for this transcription factor in the differentiation of dB4 neurons during embryonic and postnatal development. Conditional knockout of *Wt1* in these cells caused an altered population size of V0 neurons already in the developing hindbrain, leading to a decline in the respiration rate in the adults. Thereby, we confirmed and extended the previously proposed similarity between dB4 neurons in the hindbrain and dI6 neurons of the spinal cord, in terms of development and function. Ablation of Wt1+ dB4 neurons resulted in the death of neonates due to the inability to initiate respiration, suggesting a vital role for Wt1+ dB4 neurons in breathing. These results expand the role of Wt1 in the CNS and show that, in addition to its function in differentiation of dI6 neurons, it also contributes to the development of dB4 neurons in the hindbrain that are critically involved in the regulation of respiration.

## Introduction

In vertebrates, the generation of rhythmic activity (e.g., breathing and walking) is mediated by a network of neurons commonly referred to as central pattern generator (CPG) networks (Hooper, 2000). These networks are responsible for generating repetitive patterns of motor activity that do not require sensory input. However, sensory input is crucial for the refinement of CPG activity in response to external events.

**FIGURE 1:**
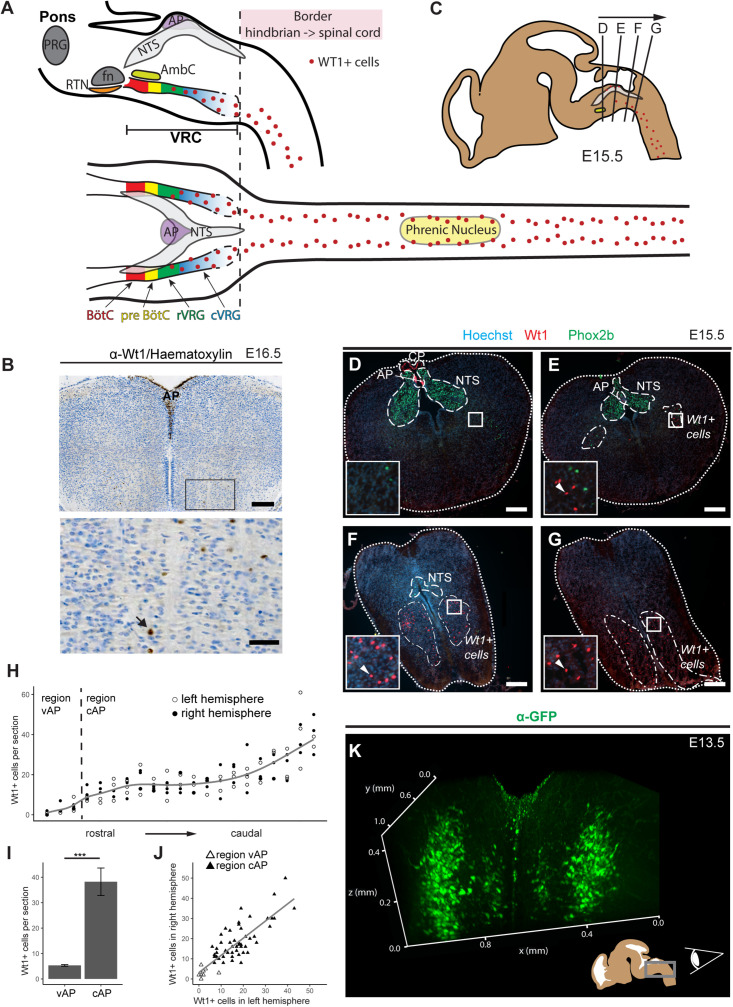
Novel domain of Wt1+ cells in the caudoventral medulla. **(A)** Sagittal and horizontal view onto respiratory related regions in brain stem and spinal cord of adult mice. The respiratory center is situated in the pons and the medulla of the mouse brain. It consists of the pontine respiratory group (PRG) and the ventral respiratory column (VRC). The VRC contains the neurons that comprise the respiratory CPG. These neurons are clustered in different compartments such as the retrotrapezoid nucleus (RTN), the Bötzinger- (BötC) and the pre-Bötzinger complex (pre-BötC), as well as the rostral and caudal ventral respiratory group (rVRG and cVRG). BötC and pre-BötC contain neurons responsible for expiration and inspiration, respectively. These neurons are modulated by the RTN that possesses sensory function for CO_2_. For inspiration, neurons in the pre-BötC innervate neurons in the rVRG and the cVRG that activate motor neurons in the phrenic nucleus of the cervical spinal cord. These motor neurons finally innervate the muscles of the diaphragm, leading to their contraction and thereby inspiration. AP: area postrema; fn: facial nucleus; AmbC: compact part of nucleus ambiguous. **(B)** Immunohistochemical analysis of Wt1 on sections of the hindbrain from E16.5 embryos reveals a region of the caudoventral medulla where *Wt1* expressing cells occur. This region is ventral to the V-shaped area postrema (AP) where non-neuronal Wt1+ cells can be found. Higher magnification image shows area in the ventral medulla where Wt1+ cells can be found. Nuclei were counterstained with hematoxylin, which shows that Wt1 localizes in the nucleus (black arrow). Orientation of the images: dorsal-up and ventral-down. Scale bar top row: 200 μm; bottom row: 50 μm. **(C)** The scheme represents a sagittal brain view demonstrating the levels of the coronal sections D, E, F, and G. Cells harboring Wt1 are depicted as red dots, whereas the region of Phox2b positive cells, which are situated in the NTS, is labeled in gray. The compact part of nucleus ambiguous (AmbC) is shown in light green for orientation. **(D)** In coronal section from rostral levels of the medulla oblongata, the localization of Wt1 is restricted to the choroid plexus (CP) and the area postrema (AP). **(E)** At a particular position ventral of the AP, where the CP is no longer observable, another population of Wt1+ cells occur in the ventral proportion of both hemispheres. **(F,G)** The area of the Wt1 populations in both hemispheres extends and enlarges in caudal direction. Sections were obtained from embryos staged E15.5. Scale bars: 200 μm. **(H)** The distribution of Wt1+ cells in coronal sections was determined along the rostrocaudal axis. The number of Wt1+ cells increases in caudal direction within the region ventral to the AP (vAP). In the region caudal to the AP (cAP), the Wt1 cell number stabilizes until it increases further caudally. Regression using generalized additive models between cell number and position along the rostrocaudal axis is shown in gray. The cell number of Wt1+ cells in every 5th coronal section (12 μm) was determined. n = 3 individuals (E15.5). **(I)** The number of Wt1+ cells in the region cAP is increased compared to the number in the region vAP. Statistical test: linear mixed model, *p*-value = 0.00000174 (***). **(J)** Linear regression between the number of Wt1+ cells in the left and right hemisphere shows correlation of the number of Wt1+ cells between the hemispheres. Statistical test: linear regression model, adjusted R^2^ value: 0.6886. **(K)** 3D reconstruction of the caudal hindbrain shows the distribution of Wt1+ cells (green). To label the cells, hindbrains from E13.5 *Wt1*^*GFP*^ embryos were used. GFP was detected using whole mount immunofluorescence staining followed by tissue clearing and light sheet microscopy. Wt1+ neurons are distributed in two lateral columns and two smaller and more medial columns. View: from caudal to rostral (as shown in the scheme).

Breathing movements are coordinated by the respiratory CPG in the brainstem (Figure 1A). The generation and regulation of the breathing rhythm is achieved by neurons concentrated in three main brainstem areas (Alheid and McCrimmon, 2008): the pontine respiratory group (PRG), the dorsal respiratory group within the nucleus of the solitary tract (NTS) and the ventral respiratory column (VRC). The latter contains the major neuron populations, clustered in different compartments that build up the respiratory CPG. Excitatory pacemaker neurons – which are primarily responsible for rhythmic inspiration – are localized in the pre-Bötzinger complex (McKay et al., 2005), which can be found in each hemisphere of the hindbrain (Gray et al., 2010). This bilateral connection allows generation of a synchronous respiratory rhythm that is required to innervate the muscles of the thorax simultaneously in each half of the body (Bouvier et al., 2010). Also involved in the regulation of respiration is the Bötzinger complex, which contains neurons that inhibit inspiration and therefore allow passive expiration during normal breathing (Ezure et al., 2003). Both the Bötzinger and pre-Bötzinger complexes project to neurons of the ventral respiratory group (VRG). The rostral and caudal ventral respiratory groups (rVRG and cVRG) encompass premotor neurons responsible for activation of inspiratory and expiratory motor neurons (MNs), respectively (Alheid and McCrimmon, 2008). The inspiratory MNs in the phrenic nucleus of the cervical spinal cord ultimately innervate the muscles of the diaphragm being the mechanical driver for inspiration. Expiration requires additional MNs and muscles such as expiratory abdominals (Abdala et al., 2009).

The high level of compartmentalization results from a distinct spatial patterning during embryonic development. This patterning and the neuronal cell fate is driven by a specific set of different transcription factors that are similar between the spinal cord and the hindbrain (Hernandez-Miranda et al., 2016). The transcription factor Lbx1, for instance, is crucial for the development of dorsal class B neurons: dB1–dB4 in the hindbrain (Sieber et al., 2007) and dI4–dI6 neurons in the spinal cord (Gross et al., 2002; Müller et al., 2002). Thus, inactivation of *Lbx1* alters the developmental program of somatosensory neurons to a more dorsal neuron phenotype associated with impairments in locomotion (Müller et al., 2002) and respiration (Pagliardini et al., 2008), respectively.

We and others have recently shown that inactivation of the Wilms tumor suppressor gene *Wt1* in the spinal cord, leads to impairments of locomotion behavior (Haque et al., 2018; Schnerwitzki et al., 2018). Wt1 was originally reported in the development and homeostasis of mostly mesoderm-derived tissues like gonads, kidneys, spleen and heart (Kreidberg et al., 1993; Herzer et al., 1999; Moore et al., 1999; Chau et al., 2011; Dong et al., 2015). Although reported to be expressed in the area postrema (AP) of the medulla oblongata (Armstrong et al., 1992; Sharma et al., 1992), Wt1 has only recently been shown to also have a function in the CNS (Haque et al., 2018; Schnerwitzki et al., 2018). It acts as a zinc finger transcription factor on the development of dI6 neurons in the spinal cord, which participate in the control of locomotion. A similarity between dI6 neurons of the spinal cord and class B neurons of the hindbrain has been proposed in terms of development (Hernandez-Miranda et al., 2016). When we looked for Wt1+ cells in the hindbrain, the question arose as to whether these cells are involved in respiration and thereby share functional similarities with dI6 neurons of the spinal cord. Our studies revealed a population of dB4 neurons expressing *Wt1* and we could further show that these cells are crucial for regulation of respiration.

## Results

### Wt1 Is Expressed in Cells of the Caudoventral Medulla

Wt1+ cells have already been reported to occur in a region of the medulla oblongata, below the fourth ventricle, called area postrema (AP) (Armstrong et al., 1992; Sharma et al., 1992). When investigating Wt1’s role during the development of dI6 neurons in the spinal cord (Schnerwitzki et al., 2018), we discovered an additional and thus far unreported expression domain of *Wt1* in the hindbrain (Figure 1A). By performing immunohistochemical staining of coronal sections through the embryonic medulla, we could confirm the presence of Wt1+ positive cells in the AP (Figure 1B). In addition, we observed further Wt1+ cells in a more ventral region of the medulla.

To examine the distribution of the Wt1+ cells in ventral parts of the developing medulla, consecutive coronal sections from embryonic mice at stage E15.5 were analyzed (Figure 1C). The nucleus of the solitary tract (NTS) was labeled by Phox2b staining for orientation within the hindbrain. When going through the sections from rostral to caudal (Figures 1D–G), Wt1+ cells were only detected at the most rostral sections in the AP and caudal parts of the choroid plexus of the fourth ventricle, but not in the ventral region of the medulla. Further caudal, the dorsal occurrence of Wt1+ cells is restricted to the AP (Figure 1E), while additional Wt1+ cells appear in two ventral areas; one per hemisphere. The extent of these areas increases in caudal direction (Figure 1F), whereas the extent of the NTS decreases until no Phox2b + cells remain (Figure 1G). This is where the dimension of the Wt1+ cell population is largest and merges with the population in the spinal cord. No discrete border was observed between both Wt1 populations – the one in the hindbrain and the one in the spinal cord.

The distribution of the ventral Wt1+ cells in both hemispheres was quantified along the rostrocaudal axis in more detail (Figure 1H). To this end, Wt1+ cells were counted on every 5^th^ consecutive coronal section. Additionally, the sections were stained for Phox2b, as a marker for the NTS, to ensure that only Wt1+ cells of the hindbrain were included in the analysis and not those of the spinal cord. The staining of Wt1+ cells in the AP was used as a reference point but were not included in our quantification; only those in the ventral medulla. The quantification revealed that the number of Wt1+ cells in the region directly ventral to the AP (vAP) increases in caudal direction. More caudally, in sections with no AP anymore, the number of Wt1+ cells is constant over a certain area before further increasing at the most caudal parts of the hindbrain. This observation prompted us to cluster the ventral Wt1+ cells into two regions; firstly the rostral part of the Wt1 population located ventral to both the AP and the NTS (vAP); then the region that includes the caudal fraction of the Wt1 cell population, still ventral of the NTS but caudoventral of the AP (cAP). Comparison of the number of ventral Wt1+ cells in both regions showed the vAP region to have significantly fewer Wt1+ cells than the cAP region (Figure 1I). Additional quantification of Wt1+ cells between the left and right hemispheres of the medulla showed that the Wt1+ cells are evenly distributed on both sides (Figure 1J).

Bearing in mind the limitations of extrapolating a spatial distribution on the basis of serial sections, a 3D reconstruction of the special distribution of Wt1+ cells in the ventral medulla was carried out using a *Wt1*^*GFP*^ reporter mouse line (Hosen et al., 2007). This mouse model has been used in previous studies (Schnerwitzki et al., 2018) by us, because it labels the soma and the major projections of Wt1+ neurons and thus adds further spatial information to the restricted nuclear localization when staining for transcription factor Wt1. After whole mount immunofluorescence staining, subsequent tissue clearing and application of light sheet microscopy, the 3D reconstruction revealed two parallel columns of Wt1+ cells in the ventral area of the medulla, one in each hemisphere (Figure 1I and Supplementary Video 1). Two minor columns were also detected, which are situated more medial. Since GFP also labels processes, we looked for projections crossing the midline. However, no commissural projections were detected at this stage (E13.5). Thus, Wt1+ cells in the ventral hindbrain appear as four domains – two major and more laterally located ones and two minor, more medially located ones.

### Temporal Distribution of Wt1+ cells in the Caudoventral Hindbrain

**FIGURE 2:**
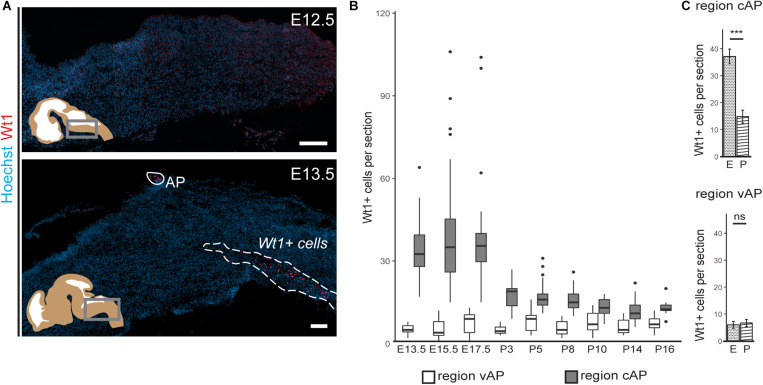
Occurrence of Wt1 in cells of the caudoventral hindbrain during development. **(A)** Immunofluorescence analyses of sagittal sections from embryonic hindbrains reveal presence of Wt1+ cells in the AP and in the caudoventral hindbrain at developmental stage E13.5. At earlier stages, no Wt1+ cells are detectable in the hindbrain. Scale bars: 100 μm. **(B)** The box plot shows the average number of Wt1+ cells that was determined for every 5th coronal hindbrain section from different embryonal and postnatal stages. The cells were categorized by their occurrence relative to the AP in a region caudal of the AP (cAP) and ventral of the AP (vAP). The number of Wt1+ cells in the region cAP is higher than in the region vAP. Postnatally, the number of Wt1+ cells decreases in the region vAP to cell numbers comparable to the region cAP. n per stage = 2–3 individuals. **(C)** The number of Wt1+ cells in sections from embryonal (E) and postnatal (P) hindbrains was compared for the region cAP and vAP. In the region cAP, the number of Wt1+ cells decreases in postnatal hindbrains compared to embryonal ones. In the region vAP, the number of Wt1+ cells is not significantly altered. n for embryonal stage = 7 individuals. n for postnatal stage = 12 individuals. Statistical test: linear mixed model, *p*-values: region vAP = 0.288 (ns = not significant), region cAP = 6.3 × 10^– 09^ (***).

To establish when Wt1+ cells first occur in the hindbrain, brains of developmental stages E11.5, E12.5 and E13.5 were analyzed (Figure 2A). While no Wt1+ cells were detected up to E12.5, a first occurrence was observed in the ventral medulla at E13.5; the same stage at which they appear in the AP.

Next, we investigated how the temporal distribution of Wt1+ cells in the ventral hindbrain changes during development (Figure 2B). The average number of Wt1+ cells in coronal section of regions both ventral and caudal to the AP was determined. While the amount of cells ventral to the AP remains constant in all investigated developmental stages (Figure 2C), more cells occur in the region caudal to the AP at later embryonal stages (E13.5–E17.5) and the cell number also varies significantly. At the postnatal stages, however, the number of Wt1+ cells decreases significantly in this region (Figure 2C). In order to exclude that the decrease in cell numbers is due to apoptosis of Wt1+ cells, we performed TUNEL-assay with hindbrain sections from newborns (Supplementary Figure 1); however, no TUNEL positive Wt1+ cells were detected (0 out of 42 examined Wt1+ cells). These findings point to a dynamic regulation of *Wt1* expression in those cells during development, dependent on their position in the hindbrain.

### Wt1 Expressing Cells in the Caudoventral Medulla Are dB4 Neurons

**FIGURE 3:**
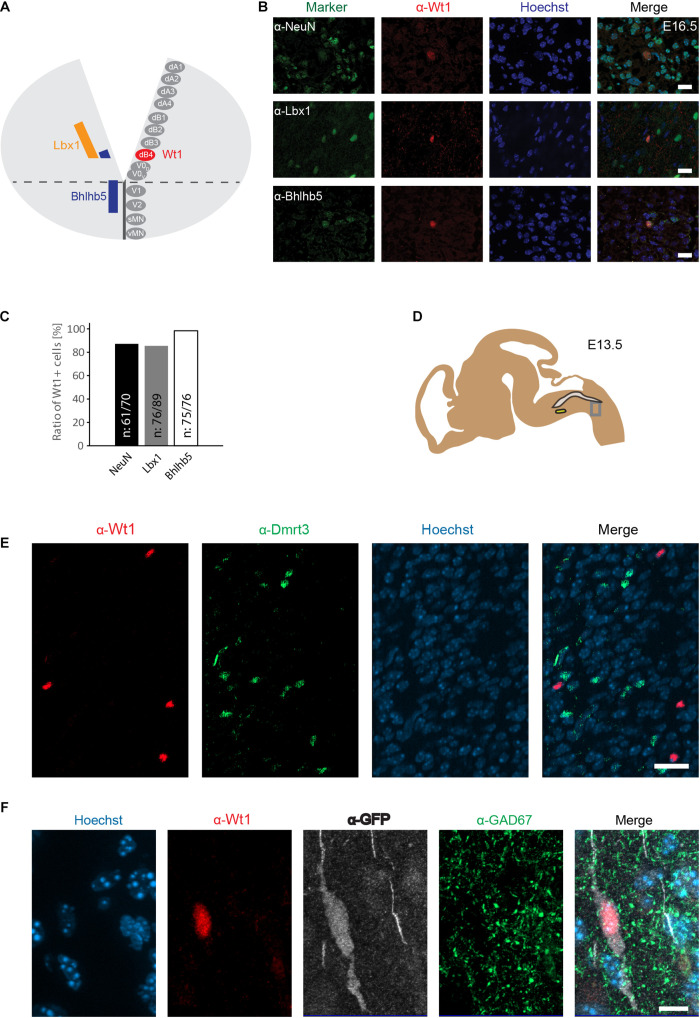
Wt1+ cells in the caudoventral medulla are dB4 neurons. **(A)** Schematic illustration of embryonic hindbrain. Neurons in the hindbrain arise from various progenitor domains surrounding the central channel: 8 dorsal neuron domains (dA1 to dB4), four ventral neuron domains (V0 to V2) and one somatic (sMN) and one visceral motor neuron domain (vMN). Lbx1 and Bhlhb5 were used as markers to determine the dB4 domain from which Wt1+ neurons (red) arise in the hindbrain. **(B)** Hindbrain sections from E16.5 embryonic mice used for immunofluorescence analyses of Wt1+ neurons with the neuronal marker NeuN and particular markers (Lbx1; Bhlhb5) present in dB4 and adjacent neuron populations. Overlapping localization of Wt1, Lbx1, and Bhlhb5 reveals dB4 origin for Wt1+ neurons. Scale bar: 20 μm. **(C)** Quantification of Wt1+ cells being positive for NeuN, Lbx1, or Bhlhb5 in hindbrains of E13.5 embryos. Number of respective cells in relation to Wt1 positive cells is given. n = 5-6 sections from one animal. **(D)** The scheme shows the area from which the image in D was taken. The NTS is labeled in gray and the AmbC is shown in light green for orientation. Developmental stage: E13.5. **(E)** Immunofluorescence analyses showed presence of Dmrt3 + cells (green) in the ventral hindbrain in proximity to Wt1+ neurons (red). No co-localization of Dmrt3 and Wt1 was observed. Scale bars: 20 μm. **(F)** Immunofluorescence analyses showed GAD67 (green) to be present in the ventral hindbrain Wt1+ neurons (red) expressing GFP (white). The occurrence of vesicular GAD67 suggests Wt1+ cells to be inhibitory, GABAergic neurons. Scale bar: 10 μm.

The similarity between spinal cord and hindbrain in terms of development, has led to the proposal that Wt1 would be a specific marker for dB4 neurons in the hindbrain, as it is for dI6 neurons in the spinal cord (Hernandez-Miranda et al., 2016; Figure 3A). Immunofluorescence analysis was performed to verify this hypothesis, using hindbrain section from E16.5 embryos (Figure 3B). Wt1+ cells in the ventral medulla were found to harbor the neuronal marker NeuN (87%), showing that these cells are neurons (Figure 3C). Moreover, the vast majority of the ventral Wt1+ neurons can also be labeled with antibodies against the transcription factors Lbx1 (85%) and Bhlhb5 (99%), that commonly occur in the most ventral Lbx1 domain that gives rise to dB4 neurons (Figure 3C). The Wt1+ cells in the AP were neither positive for Lbx1 nor Bhlhb5 and therefore not included in the quantification of the ratio. Thus, our data confirms Wt1 to be a marker for at least a subpopulation of dB4 neurons (Figure 3A).

A subpopulation of dI6 neurons in the spinal cord is characterized by the presence of the transcription factor Dmrt3. This subpopulation overlaps in part with Wt1+ cells, giving rise to a small Dmrt3/Wt1 double-positive population of dI6 neurons (Andersson et al., 2012). Dmrt3 has also been suggested but not yet shown to occur in dB4 neurons (Hernandez-Miranda et al., 2016). Immunofluorescence analyses revealed that Dmrt3 + cells are indeed present in the ventral hindbrain and situated in close vicinity to the Wt1+ neurons (Figure 3D). However, no co-localization was observed between Wt1 and Dmrt3 (Figure 3E).

Given that Wt1+ dI6 neurons in the spinal cord have been described as inhibitory neurons (Haque et al., 2018), we tested whether Wt1+ cells in the hindbrain of *Wt1*^*GFP*^ newborn mice have inhibitory properties by co-staining with GAD67, a marker for GABAergic neurons. The immunofluorescent analyses revealed occurrence of vesicular GAD67 in Wt1+ cells, suggesting Wt1 as a marker for inhibitory dB4 neurons.

### Alterations in Neuron Composition Upon Wt1 Inactivation

**FIGURE 4:**
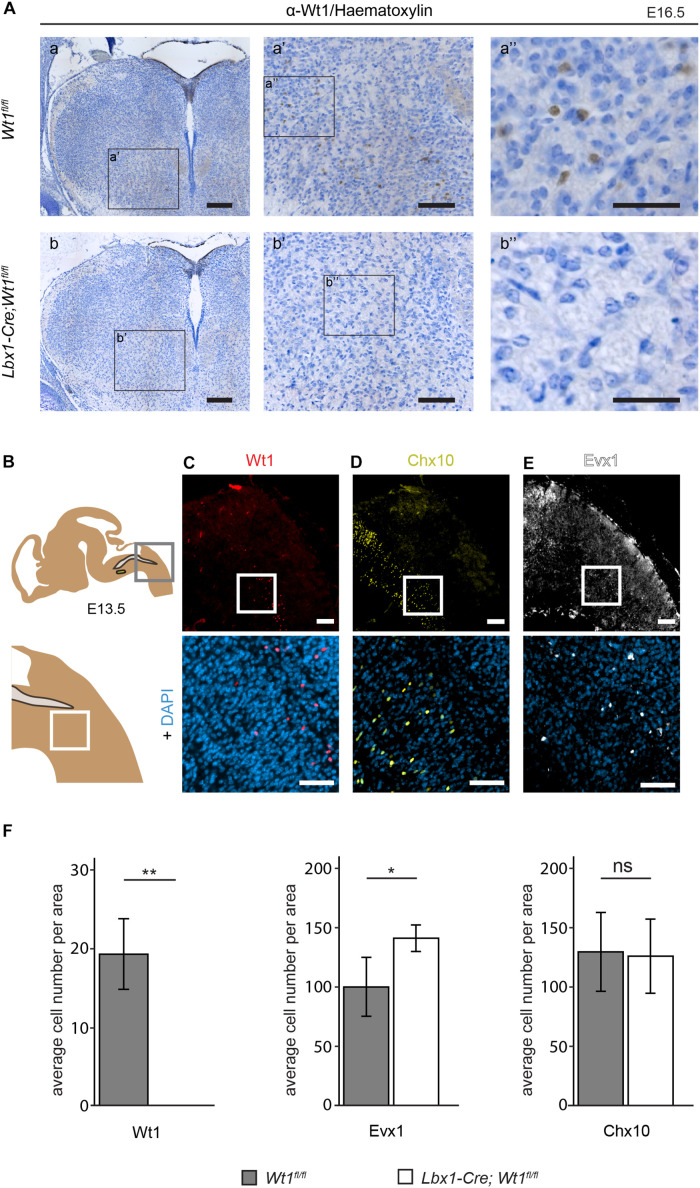
Neuron composition in conditional *Wt1* knockout embryos. **(A)** Immunohistochemical analyses of Wt1 on hindbrain sections from *Lbx1-Cre;Wt1^*fl/fl*^* embryos (E16.5) and control animals were used to verify deletion of *Wt1* in the ventral medulla of *Lbx1-Cre;Wt1^*fl/fl*^* animals. To ensure that the same hindbrain region of *Lbx1-Cre;Wt1^*fl/fl*^* and control embryos was examined, the non-neuronal Wt1+ cells found in the V-shaped AP were used as landmarks. Higher magnification images (a’; a” and b’; b”) show that Wt1 is not detectable in the ventral medulla of *Lbx1-Cre;Wt1^*fl/fl*^* compared to control animals. Orientation of the images: dorsal-up and ventral-down. Scale bar left column: 100 μm; middle column: 100 μm; right column: 50 μm. **(B)** Upper panel: Scheme of embryonal hindbrain with the NTS labeled in gray and the AmbC shown in light green for orientation. Area in gray frame shows the region in which the Wt1, Chx10 and Evx1 + cells of Wt1 knockout and control mice were counted. Lower panel: magnification. **(C–E)** Upper panel: immunofluorescence staining of sagittal embryonic hindbrain sections (E13.5) show the area harboring the Wt1, Evx1, and Chx10 + cells that were used for quantification in panel F. Lower panel: higher magnification was used to confirm nuclear localization of the respective staining for Wt1, Evx1, and Chx10 + cells. Wt1+ neurons are distributed in the ventral part **(C)**. The population of Chx10+ cells extends further rostral and dorsal. **(D)**. Evx1 labeled cells are diffusely distributed over the hindbrain. **(E)**. Scale bars: 100 μm. **(F)** Quantification of the average cell number of Wt1, Evx1 and Chx10 + cells, in those areas of the hindbrain mentioned in B, from conditional *Wt1* knockout embryos (*Lbx1-cre;Wt1^*fl/fl*^*) reveals an absence of Wt1+ neurons compared to control individuals. The number of Chx10 + cells per area remains constant upon *Wt1* knockout but the number of cells labeled by Evx1 per area increases. Statistical test: linear mixed model, *p*-values/n-number: p(Wt1) = 0.00407 (**)/*n* = 3, p(Evx1) = 0.0116 (*)/*n* = 5, p(Chx10) = 0.85528 (ns = not significant)/*n* = 3.

Having determined the spatial and temporal distribution of the Wt1+ neurons, we aimed to apply the conditional *Wt1* knockout mouse model *Lbx1-Cre;Wt1^*fl/fl*^* to reveal possible functions of Wt1 in the dB4 neurons. Embryos at stage E16.5 were used for immunohistochemical analyses of Wt1 presence in the ventral medulla, to verify the conditional *Wt1* deletion. *Lbx1-Cre;Wt1^*fl/fl*^* embryos did not show any Wt1+ neurons in the ventral region of the medulla, where the cells occur in control animals (Figure 4A). The Wt1+ cells in the AP and the Wt1+ cells in the choroid plexus were not affected by the conditional *Wt1* knockout in *Lbx1-Cre;Wt1^*fl/fl*^* animals. This shows that neither of those populations expresses *Lbx1* and thus are not dB4 neurons; so, this mouse line allows specific deletion of *Wt1* in the neurons of the ventral medulla already at embryonic stage.

Having observed alterations in the neuronal composition of the developing spinal cord upon deletion of *Wt1* (Schnerwitzki et al., 2018), we intended to verify if the extent of several neuronal subtypes is changed in the hindbrain of *Wt1* knockout embryos – focusing on those ventral neuron subtypes that we had found to be altered in the spinal cord, namely Evx1+ V0 neurons and Chx10+ V2a neurons.

The unequal distribution of Wt1, Chx10, and Evx1 + cells in the hindbrain means their respective cell numbers depend strongly on the orientation (coronal or sagittal) and the spatial level of the section. Therefore, cell numbers were normalized to a defined area (908 μm × 908 μm) in sagittal sections. This area encompasses the caudal part of the section with the border between spinal cord and hindbrain (bottom right corner) and the dorsal edge of the medulla (top left corner) (Figure 4B - upper panel). As shown before, Wt1+ neurons are distributed ventral of this area in a stripe-like fashion (Figure 4C). There are no Wt1+ cells rostral of the defined area. Hence, only sections through the respiratory columns including at least the whole cVRG have been taken under consideration. Chx10 and Evx1 + cells occur in close proximity to Wt1+ cells in the ventral hindbrain (Figures 4D,E). The population labeled by Chx10 extends further rostral and dorsal than the Wt1 cohort (Figure 4D). Besides, the Evx1 + cells are enriched in the ventral area but show an additional diffuse distribution over the whole hindbrain (Figure 4E). In recognition of this, all numbers are based on cells in the vicinity of both columns where the Wt1+ neurons occur.

The quantitative analysis of the cell number in that defined area of each section confirms the absence of Wt1+ cells in the ventral region of E13.5 *Lbx1-Cre;Wt1^*fl/fl*^* embryos (Figure 4F). As the Wt1+ cells of the AP turned out not to be dB4 neurons, they too were not considered for counting. The determination of the number of Evx1 and Chx10 + cells revealed a significant increase in the amount of Evx1 + cells in the respective area upon deletion of *Wt1*, while the number of Chx10 + cells did not change at developmental stage E13.5. This decline in the number of Wt1+ dB4 neurons and the concomitant increase in the amount of Evx1 + cells, might point to a change in the developmental fate from dB4 neurons into V0 neurons upon loss of Wt1.

### Wt1+ dB4 Neurons Are Necessary for Respiration

**FIGURE 5:**
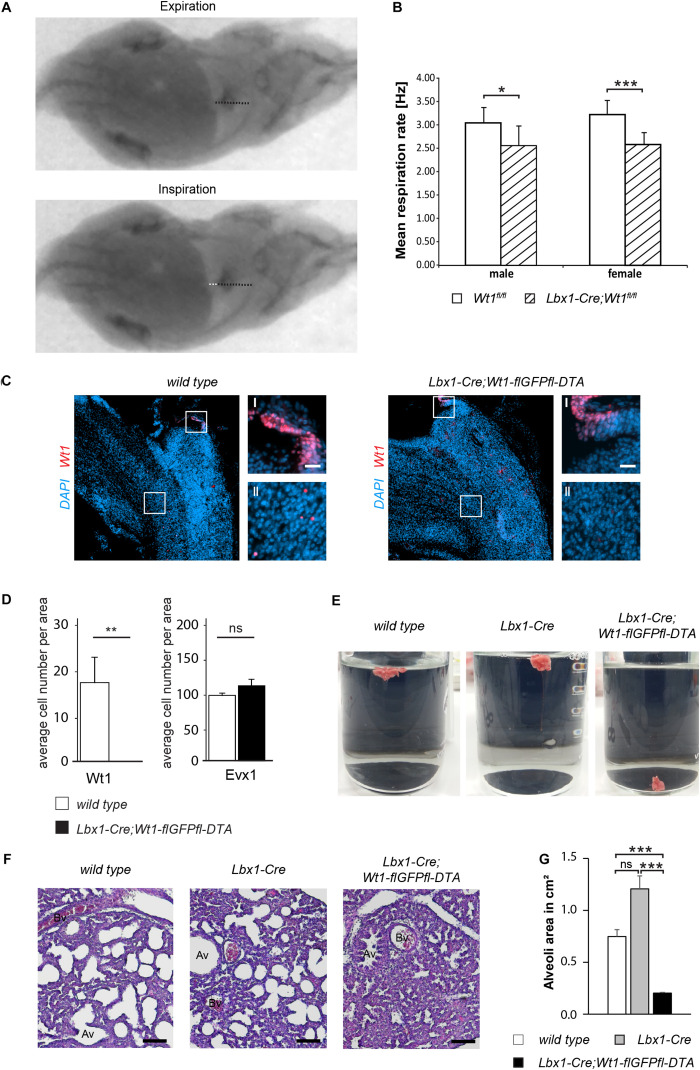
*Wt1* + dB4 neurons are crucial for respiration. **(A)** Respiration rate was determined by recording X-ray radiographs of mice at rest. The movement of the diaphragm over time was analyzed (white dotted line in bottom image) and the mean respiration rate was calculated as the number of inspirations per second. Orientation of mice: ventral view with posterior on the left and anterior on the right. **(B)** The mean respiration rate was determined for *Lbx1-Cre;Wt1^*fl/fl*^* mice and control animals. In both male and female *Lbx1-Cre;Wt1^*fl/fl*^* mice the respiration rate is significantly decreased compared to controls. For both sexes *n* = 10 *Wt1*^*fl/fl*^ control; *n* = 10 *Lbx1-Cre-Cre;Wt1^*fl/fl*^*. Data are expressed as mean ± SD. Significance level: ***P < 0.001; *P < 0.05 (according to Student’s *t*-test). **(C)** Immunohistochemical analyses of Wt1 on hindbrain sections from *Lbx1-Cre;Wt1-flGFPfl-DTA* embryos (E13.5) and wild type animals verify ablation of *Wt1* + cells in the ventral medulla of *Lbx1-Cre;Wt1-flGFPfl-DTA* embryos. To ensure that the same hindbrain region of *Lbx1-Cre;Wt1-flGFPfl-DTA* and control embryos was examined, the non-neuronal Wt1+ cells found in the AP were used as landmarks (magnification I). Higher magnification images (magnification II) show that Wt1+ cells are not detectable in the ventral medulla of *Lbx1-Cre;Wt1-flGFPfl-DTA* compared to wild type animals. Region is situated according to the upper panel of Figure 4B. Scale bars overview: 100 μm. Scale bars magnification: 20 μm. **(D)** Quantification of the average cell number of Wt1 and Evx1 + cells in those areas of the hindbrain mentioned in 4B from Wt1+ cell ablation embryos (*Lbx1-cre;Wt1-flGFPfl-DTA*) reveals an absence of Wt1+ neurons compared to control individuals. The number of cells labeled by Evx1 per area remains constant upon Wt1+ cell ablation. Statistical test: linear mixed model, *p*-values/n-number: p(Wt1) = 0.00445 (**)/*n* = 3, p(Evx1) = 0.183 (ns = not significant)/*n* = 3. **(E)** Neonates of *Lbx1-Cre;Wt1-flGFPfl-DTA* exhibit respiratory failure. Lung hydrostatic tests show sinking of lungs from *Lbx1-Cre;Wt1-flGFPfl-DTA* neonates as they have never inflated their lungs properly after birth; in contrast to lungs from control wild type and *Lbx1-Cre* animals whose lungs are floating due to inspiration and inflation of lungs with air *n* = 6. **(F)** Histological sections of lungs from control animals and *Lbx1-Cre;Wt1-flGFPfl-DTA* neonates were stained with eosin and hematoxylin. The alveoli of control animals are inflated with air. In *Lbx1-Cre;Wt1-flGFPfl-DTA* neonates, the alveoli have never inflated after birth (Av – alveolus; Bv – blood vessel). **(G)** The mean alveoli area was determined on the bases of eosin- and hematoxylin-stained lung sections of the respective genotypes. Due to the absent onset of respiration in *Lbx1-Cre;Wt1-flGFPfl-DTA* neonates, their mean alveoli area was significantly smaller compared to *Lbx1-Cre* and wild type litter mates. Data are expressed as mean ± SEM. Significance level: ***P < 0.001 (according to Student’s *t*-test); ns = not significant; *n* = 28–29 alveoli from one animal per genotype.

To analyze the putative involvement of the Wt1+ dB4 neurons in respiration, adult *Lbx1-Cre;Wt1^*fl/fl*^* were examined for their respiration rate. This was determined by recording X-ray radiographs of mice at rest in order to count the number of inspirations by observing the movement of the diaphragm (Supplementary Video 2; Figure 5A). The mean respiration rate was calculated as the number of inspirations per second (Figure 5B). For *Lbx1-Cre;Wt1^*fl/fl*^* mice, the mean respiration rate was significantly decreased compared to control animals. Although these changes in respiration rate were observed for both sexes, the effect was more pronounced in females. Thus, the alterations that we observed in conditional *Lbx1-Cre;Wt1^*fl/fl*^* mouse embryos, namely the loss of Wt1 in dB4 neurons and the concomitant increase in the amount of Evx1 + neurons, manifests itself as a change in respiration rate in adult mice.

Considering that the respiratory phenotype in the *Lbx1-Cre;Wt1^*fl/fl*^* mice might be a result of either the deletion of *Wt1* or the increase of the Evx1 + cell number, we decided to examine the role of the Wt1+ dB4 neurons by ablating these cell and thereby avoiding the increase in Evx1 cell number. As reported earlier (Schnerwitzki et al., 2018), *Lbx1-Cre;Wt1-GFP-DTA* mice show ablation of Wt1- and Lbx1-co-expressing cells in the spinal cord due to the diphtheria toxin subunit A (*DTA*), which is expressed from the endogenous *Wt1* locus after Cre-mediated excision of a *GFP* cassette harboring a translational STOP codon. Our study detected an ablation of Wt1+ cells in the embryonic hindbrain. As for the conditional knockout of Wt1, only the Wt1+ dB4 neurons in the ventral medulla were affected, whereas the Wt1+ cells in the AP and the choroid plexus could still be observed (Figure 5C). When quantifying the number of Wt1+ cells in the ventral medulla of *Lbx1-Cre;Wt1-GFP-DTA* embryos at stage E13.5, as was done for the *Lbx1-Cre;Wt1^*fl/fl*^* embryos, we could not detect any Wt1+ cells in the analyzed sections (Figure 5D). Needing to verify whether a change in the developmental fate from dB4 neurons into V0 neurons upon loss of Wt1 occurs, as observed in the *Lbx1-Cre;Wt1^*fl/fl*^* embryos, we determined the number of Evx1 + cells upon ablation of Wt1+ cells. Contrary to the conditional *Wt1* knockout, the number of Evx1 + cells was not altered when ablating Wt1+ cells – suggesting that the knockout of *Wt1* leads to a change in the developmental fate of dB4 neurons that is not possible when those cells are ablated.

Monitoring of *Lbx1-Cre;Wt1-flGFPfl-DTA* animals at birth, showed them to be vital, as assessed by body movements after non-noxious stimuli of the skin. However, *Lbx1-Cre;Wt1-flGFPfl-DTA* neonates did not exhibit gasping after birth, which would then turn into abdominal breathing, as seen for control littermates. *Lbx1-Cre;Wt1-flGFPfl-DTA* did not show contraction of the abdominal wall and became cyanotic; eventually no longer responding to stimuli, leading to death.

Lung hydrostatic tests and histological analyses of the lung were performed, in order to verify the phenotype of *Lbx1-Cre;Wt1-flGFPfl-DTA –* which is the incapability of neonates to initiate proper respiration (Figure 5E). In wild type animals, the lung inflates during the first breaths. When the lung is transferred into water, it floats due to the inflation with air. This effect was also seen with lungs from *Lbx1-Cre* controls (4/4). Newborn *Lbx1-Cre;Wt1-flGFPfl-DTA* animals did not breathe and never inflated their lungs properly; as a result, their lungs sank (2/2).

The absent inflation of the lung was also detected histologically (Figure 5F). The alveoli of wild type and *Lbx1-Cre* animals were inflated, seen by their round shape. Alveoli from *Lbx1-Cre;Wt1-flGFPfl-DTA* neonates were uninflated and thus showed a reduced mean alveoli area (Figure 5G). These morphological alterations confirm that *Lbx1-Cre;Wt1-flGFPfl-DTA* neonates are impaired in developing proper respiration at birth.

These data show that the Wt1+ dB4 neurons in the medulla are involved in regulating respiration and that deletion of *Wt1* leads to a decreased respiration rate. Moreover, the existence of these Wt1+ dB4 neurons is a prerequisite for newborn animals to initiate respiration, as ablation of these cells leads to death at birth.

## Discussion

In this study we show that *Wt1* is expressed in dB4 neurons during mouse embryonic and postnatal development. These Wt1+ neurons are located in one major and one minor column in each hemisphere of the medulla. If *Wt1* is deleted in dB4 cells at an embryonic stage, the neuronal composition of the hindbrain is altered. The changes also have late effects in that the respiration rate of adult mice with *Wt1* inactivation in dB4 neurons is decreased. More severe than *Wt1* deletion is the loss of the Wt1+ dB4 neurons in the hindbrain, as these cells are indispensable for breathing.

The *Lbx1-Cre;Wt1-flGFPfl-DTA* neonates show ablation of cells expressing both *Wt1* and *Lbx1*. Surprisingly, the newborns die immediately after birth, provoking the notion that cells are depleted which are essential for life. Histological analyses showed impairments of *Lbx1-Cre;Wt1-flGFPfl-DTA* neonates to developing proper respiration at birth, pointing to the fact that cells expressing both *Wt1* and *Lbx1* are involved in establishing respiration. Besides a shared expression of *Lbx1* and *Wt1* in the dB4 neurons of the medulla, both genes have been reported to be co-expressed in dI6 neurons of the spinal cord (Haque et al., 2018; Schnerwitzki et al., 2018), as well as in cells of the heart and diaphragm (Chao et al., 2011; Dingemann et al., 2011). Of those organs, the diaphragm too is essential for respiration. The question arises as to whether the inability to initiate respiration in *Lbx1-Cre;Wt1-flGFPfl-DTA* neonates might also be due to ablation of cells in the diaphragm. Although *Lbx1* and *Wt1* are expressed in the diaphragm, the expression pattern of both is mutually exclusive. The former is expressed in myogenic cells (Chao et al., 2011), whereas expression of *Wt1* has been described in non-muscular components of the developing pleural mesothelium (Dingemann et al., 2011). This implies that ablation of the Lbx1 + and Wt1+ dB4 neurons in the medulla seems causative for the neonatal lethality.

A similarity between Wt1+ dB4 neurons in the hindbrain and Wt1+ dI6 neurons of the spinal cord has been proposed on the basis of certain markers occurring in both neuronal types during development (Hernandez-Miranda et al., 2016). As the transcription factors we used to verify that Wt1 cells belong to the dB4 population are also expressed by the late born neuron type dBLa, we could not exclude that a fraction of Wt1+ cells might be a part of the dBLa population that arise from the dB1 progenitor domain (Hernandez-Miranda et al., 2016).

We were able to confirm the similarity of Wt1 cells in the hindbrain to the dI6 neurons of the spinal cord, not only on the level of the occurrence of common transcription factors but also on the behavioral level. Thus, tissue-specific knockout of *Wt1* in dB4 neurons using the *Lbx1-Cre;Wt1^*fl/fl*^* mouse model showed functional similarities to the dI6 neurons in the spinal cord. Whilst these animals were vital, they did show a decreased respiration rate at rest comparable to the decline in stride frequency in the animals with *Wt1* deletion in dI6 neurons (Schnerwitzki et al., 2018). The decreased respiration rate resembles the slower respiratory rhythm observed in constitutive, homozygous *Lbx1* knockout embryos. Pagliardini et al. (2008) reported that *Lbx1* expression in particular neurons of the medulla is necessary to avoid respiratory defects. Here, a proper rhythm was shown to be generated by the pre-Bötzinger cells in these embryos at E18.5. Yet this rhythm is not transmitted properly to the motor neurons in the phrenic nucleus of the spinal cord; consequently, the embryos die at birth due to respiratory failure. That the deletion of *Lbx1* leads to death of neonates might be explained by the fact that more neurons in the hindbrain express *Lbx1* than *Wt1*. The knockout of *Lbx1* thus affects more neurons involved in various motoric and sensory tasks. Heterozygous *Lbx1* knockout animals are not affected, remaining vital and exhibiting normal respiration.

The similarity in development between Wt1+ dB4 neurons and dI6 neurons also becomes apparent at the level of the cellular consequences of *Wt1* deletion and ablation. As our work shows, the number of Evx1 + cells increases upon *Wt1* knockout already at developmental stage E13.5. The Evx1 + neurons in the hindbrain derive from the V0 progenitor domain (Bouvier et al., 2010) and give rise especially to excitatory premotor neurons in the rVRG (Wu et al., 2017). That increase in the number of Evx1 + V0 neurons was also observed in embryonic spinal cord, where a fate change from Wt1+ neurons to V0-like neurons has been suggested when *Wt1* is deleted (Schnerwitzki et al., 2018). Further, the ablation of Wt1+ dB4 neurons in the medulla resembles the effects of ablating dI6 neurons in the spinal cord. In both interneuron populations, the number of Evx1 + neurons remains unaltered upon ablation of Wt1+ neurons. Thus, cells destined to become Wt1+ dB4 neurons might change their fate into Evx1 + neurons to compensate for the knockout of *Wt1*. If the Wt1+ neurons are ablated, there is also no compensation that would lead to an increase in the number of Evx1 + neurons.

Another neuronal population whose number was found to be altered in the spinal cord due to a knockout of *Wt1* is the population of Chx10 + V2a neurons. In the medulla, these cells have been reported to be situated in the ventral respiratory column and shown to be necessary to maintain the frequency and regularity of breathing in neonatal (Crone et al., 2012), but not in adult mice (Jensen et al., 2019). Although we cannot exclude changes in Chx10 + cell numbers in rostral parts of the medulla, which contain Chx10 + cells but were not counted in this study, the number of Chx10 + neurons in the caudal medulla is not changed in *Wt1* knockout embryos at stage E13.5. This is in line with findings in the spinal cord, where the number of Chx10 + neurons does not alter at E12.5 and only showed a decrease upon *Wt1* knockout at E16.5. Due to technical reasons, we were unable to determine the number of Chx10 + neurons in the respective area of the caudal medulla at E16.5.

In general, the behavioral and cellular studies reveal that Wt1+ dB4 neurons in the hindbrain are involved in neuronal CPG circuits responsible for rhythmic movements of the respiratory musculature, as we have reported for the Wt1+ dI6 neurons in the spinal cord. What we cannot say at this point is whether both interneuron subtypes fulfill the same functions. Although *Wt1* deletion leads to phenotypes associated with altered neuronal composition and a slowed rhythm of movements in both respiration and locomotion, it is not possible to link the common phenotype to a common function. This is mainly due to the different pattern generating mechanisms, meaning a left-right alternating rhythm in locomotion and a synchronous rhythm in respiration.

Since the deletion of *Wt1* as well as the ablation of the Wt1+ dB4 neurons are both associated with defects in respiration and Wt1+ neurons are to be found in columns in the caudal region of the medulla, the Wt1+ cells likely belong to the caudal portion of the ventral respiratory column – in particular the cVRG (Figure 1A). The cVRG is not well characterized in terms of spatial dimension and cellular composition. However, it harbors some cells described for their involvement in respiration (Smith et al., 2013). Mostly, expiratory premotor neurons and third order neurons of motor neurons in the phrenic nucleus are located in the cVRG (Dobbins and Feldman, 1994). Since our data suggest that Wt1+ dB4 neurons of the hindbrain are presumably inhibitory due to the occurrence of GAD67 in these cells, the question remains as to how Wt1+ dB4 cells contribute functionally to respiration. To functionally confirm the inhibitory character and to incorporate the cells into the respiratory CPG, the electrophysiological properties of the Wt1+ dB4 neurons have to be determined by applying the same *Wt1*^*GFP*^ mouse model used to label the Wt1+ neurons in the hindbrain.

Taken together, the reports from the *Lbx1* knockout and the findings shown in our study suggest that the dB4 neurons in the cVRG, which express both *Lbx1* and *Wt1*, are part of the respiratory CPG and crucial for breathing. As suggested for cVRG neurons (Smith et al., 2013), they seem to transmit the rhythmic signals generated in the pre-Bötzinger complex to the motor neurons in the phrenic nucleus that innervate the muscles of the diaphragm. Deletion of either *Wt1* or *Lbx1* as reported earlier (Pagliardini et al., 2008), results in changes in the developmental fate and therefore to an altered transmission of these vital signals that are associated with a decreased respiratory rhythm. Ablation of Wt1+ dB4 neurons seems to “cut the cord” between the rhythm generating pre-Bötzinger complex and motor neurons. As a consequence, animals without expression of Wt1 in dB4 neurons survive but breathe more slowly, whereas ablation of Wt1+ dB4 neurons is far more severe and leads to the death of the newborns due to the incapacity to inflate their lungs. Although this is the most likely explanation of the phenotypes in both mouse models, there exists the possibility that spinal Wt1 neurons might also contribute to the defects observed, as described earlier (Ghali, 2018).

To prove the putative transmitting position of the Wt1+ dB4 neurons, it will be necessary to verify the proper function of the rhythm generating pre-Bötzinger complex in *Lbx1-Cre;Wt1-flGFPfl-DTA* embryos at birth, by using calcium imaging and electrophysiological recordings. Additionally, a putative loss of motor neuron output of the phrenic nerve, which innervates the muscles of the diaphragm, could be detected by recording currents at the ventral roots of the cervical level C3–C5 of the spinal cord.

In sum the results obtained from our work confirm not only the similarity between the development and function of dB4 and dI6 neurons in the hindbrain and spinal cord, but also reveal the thus far undescribed necessity for Wt1+ dB4 neurons for respiration and thereby their indispensability for life.

## Materials and Methods

### Mouse Husbandry

All mice were bred and maintained in the Animal Facility of the Leibniz Institute on Aging – Fritz Lipmann Institute (FLI), Jena, Germany, according to the rules of the German Animal Welfare Law (Animal licenses: J-SHK-2684-04-08-01/14, J-SHK-2684-04-08-02/14, TG/J-0002858/A, TG/J-0003616/A, TG/J-0003681/A, 22-2684-04-03-004/14, 22-2684-04-03-049/16). Sex- and age-matched mice were used. Animals were housed under specific pathogen-free conditions (SPF), maintained on a 12 h light/dark cycle and fed with mouse chow and tap water *ad libitum*. *Wt1*^*fl/fl*^ mice were maintained on a mixed C57B6/J x 129/Sv strain. *Wt1*^*GFP*^ mice (Hosen et al., 2007) were maintained on a C57B6/J strain. Conditional *Wt1* knockout mice were generated by breeding *Wt1*^*fl/fl*^ (Gebeshuber et al., 2013) to *Lbx1-Cre;Wt1^*fl/fl*^* mice (Sieber et al., 2007). To generate mice with Wt1 ablated cells, *Wt1-flGFPfl-DTA* mice (Schnerwitzki et al., 2018) were bred with *Lbx1-Cre* mice. Control mice were sex- and age-matched littermates (wild type or *Wt1*^*fl/fl*^). For plug mating analysis, females of specific genotypes were housed with males of specific genotypes and were checked every morning for the presence of a plug. For embryo analysis, pregnant mice were sacrificed by CO_2_ inhalation at specific time points during embryo development and embryos were dissected. Typically, female mice between 2 and 6 months were used for breeding.

### Analysis of Respiration Rate of Adult Mice at Rest

10 animals per sex and genotype (*Lbx1-Cre;Wt1^*fl/fl*^* and *Wt1*^*fl/fl*^ control) were used. We recorded the respiration rate of adult mice (age: 10–16 weeks) using high-resolution X-ray fluoroscopy (biplanar C-arm fluoroscope Neurostar, Siemens AG, Erlangen, Germany). The X-ray system operates with high-speed cameras and a maximum spatial resolution of 1536 dpi × 1024 dpi. A frame frequency of 500 Hz was used. A normal-light camera operating at the same frequency and synchronized to the X-ray fluoroscope was used to document the entire trial from the lateral perspective. Respiration rate was determined by recording the movement of the diaphragm of mice at rest. The mean respiration rate was calculated for each mouse as the number of inspirations per second. Group means were calculated from the means of the 10 animals. Student’s t-test was computed to determine significance of the differences between the means of *Wt1*^*fl/fl*^ and *Lbx1-Cre;Wt1^*fl/fl*^* animals.

### Lung Hydrostatic Test

Lung hydrostatic test was performed with lungs from *Lbx1-Cre;Wt1-flGFPfl-DTA* neonates to determine whether lungs had been inflated after birth. Therefore, the entire lung was removed from the thorax and subsequently transferred into water. Floating of the lung pointed to proper inflation with air. Sinking of the lung implied absence of air therein. The relative number of floating lungs was determined taking into account the respective genotypes.

### Hematoxylin and Eosin Staining

The tissue was processed using an automated slide stainer (Leica, Wetzlar/Germany), with the following program: paraffin sections were deparaffinized with xylene 2x for 10 min, followed by a series of rehydration steps using a decreasing gradient of ethanol. The tissue was stained in eosin for 2 min followed by washing in water, further followed by hematoxylin staining for 1.5 min. Stained sections were dehydrated using an increasing gradient of ethanol and cleared in xylene. Finally, slides were mounted using xylene-based mounting medium. Microscopy images from eosin- and hematoxylin-stained lung sections of the respective genotypes were the basis for determining the mean alveoli area using ImageJ. Images were converted into 8-bit binary images by applying default automatic thresholding with black objects on white background. The area of the object was determined using the Particle Analyzer function with the following settings: size range between 0.02 and infinity and circularity between 0.12 and 1. Objects on edges were not included.

### Immunohistochemistry Staining on Sections

Paraffin sections were deparaffinized as described above. To retrieve antigens, slides were incubated in sub-boiling sodium citrate buffer for 30 min. During the incubation, the temperature was continuously checked to be between 98°C–100°C. After antigen retrieval, slides were cooled to RT then washed with H_2_O. To permeabilize the tissue and saturate endogenous peroxidases, the slides were incubated in methanol with 0.3% hydrogen peroxide for 20 min. After washing with PBS, the sections were incubated with the primary antibody (Wt1 1:50, Agilent -Dako, Santa Clara, California, United States) diluted in PBS at 4°C overnight. The next day, the slides were washed with PBS and incubated with the polymeric horseradish peroxidase (HRP)-conjugated secondary antibody for 30 min. After washing with PBS, the slides were incubated with 3,3’-Diaminobenzidine (DAB) solution for 6 min. The staining intensity was checked with the microscope and finally stopped with water. Counterstaining was performed with hematoxylin and the slides mounted using xylene-based mounting medium.

### Immunofluorescence Staining on Sections

Embryonic and postnatal brains were dissected and either frozen unfixed after 15 min dehydration with 20% sucrose (in 50% TissueTec/PBS) (post-fix), or fixed for 75 min in 4% paraformaldehyde in PBS (pre-fix). Pre-fixed tissue was cryo-protected in 10%, 20% and 30% sucrose (in PBS) before freezing in cryo-embedding medium (Neg-50 - Thermo Scientific, Kalamazoo, United States). Post- and pre-fix samples were sectioned (12 μm). Post-fixed samples were fixed for 10 min after sectioning and washed with 2% Tween in PBS (PBS-T). For pre-fixed samples, antigen retrieval was performed by incubation in sub-boiling 10 mM sodium citrate buffer pH6.0 for 30 min. After blocking with 10% goat serum and 2% BSA in PBS-T (post-fix), or with 0.1% TritonX 100, 1% donkey serum or goat serum in PBS (prefix), sections were incubated with primary antibodies (in blocking solution) using the following dilutions: Chx10 1:100 (Abcam, Cambridge, United Kingdom, ab16141, sheep), Dmrt3 1:5000 (custom made (Andersson et al., 2012), guinea pig), Evx1 1:1000 (Developmental Studies Hybridoma Bank, University of Iowa, Iowa City, IA, United States99.1-3A2-s, mouse), GAD67 1:100 (abcam, Cambridge, United Kingdom, ab26116, mouse), GFP 1:1000 (abcam, Cambridge, United Kingdom, chick, ab13970), NeuN, 1:500 (Merck, Darmstadt, Germany, MAB377, mouse), Phox2b 1:500 (Santa Cruz Biotechnology, Inc., Santa Cruz, California, United States, sc-376997, mouse), Wt1 1:100 (Santa Cruz Biotechnology, Inc., Santa Cruz, California, United States, sc-192, rabbit), Wt1 1:1000 (abcam, Cambridge, United Kingdom, ab15249, rabbit). Secondary antibodies (Alexa Fluor secondary antibodies, Thermo Fisher Scientific, Waltham, Massachusetts, United States) were applied according to species specificity of primary antibodies. Hoechst was used to stain nuclei.

### Whole-mount Immunofluorescence Staining and Tissue Clearing

Samples were treated consecutively with 75%, 50%, and 25% methanol, for rehydration. After washing with PBS-T, antigen retrieval was performed as mentioned above and samples then washed with PBS-T and incubated with blocking solution consisting of 5% NGS, 1% DMSO, and 0.5% Triton X 100 diluted in PBS. Whole-mount tissue was incubated with GFP primary antibodies (diluted 1:500 in blocking solution) at 4°C for at least 24 h. Samples were washed meticulously with PBS-T for another 24 h before incubation with secondary antibodies for at least 24 h. Following washing with PBS-T 3 times in 2 h, Hoechst (1:100) was added to the sample for overnight incubation before samples underwent a final washing with PBS-T, as before. For subsequent clearing of the tissue, the whole-mount samples were transferred into Sca*l*eA2 solution (4M Urea, 10%Glycerol, 0.1%Triton; pH 7.7) (Hama et al., 2011) and incubated at 4°C for 10 months. The level of clearing was examined every 2nd month and the Sca*l*eA2 solution replaced with a fresh one.

### TUNEL-Assay

To detect apoptosis *in situ*, TUNEL assay was performed prior to antibody binding. Slides were incubated with TUNEL reaction solution (1x Reaction Buffer TdT and 15 U TdT in ddH_2_O from Thermo Scientific; 1 mM dUTP-biotin from Roche) at 37°C for 1 hour and washed afterward with PBS.

### Imaging and Picture Processing

Fluorescent images were acquired with a Zeiss Axio Imager and a Zeiss Axio Observer Z1 equipped with an ApoTome slider for optical sectioning (Zeiss, Germany). Cleared and whole-mount stained specimen were imaged with a light sheet microscope (Lightsheet Z1, Zeiss, Germany) enabled for dual side illumination and equipped with a 20x detection objective suitable for clearing methods with refractive index of 1.38 (CLR Plan-Apochromat Corr nd = 1.38 VIS-IR, numerical aperture = 1.0, working distance = 5,6 mm). For image processing which consisted of dual side fusion, three-dimensional reconstruction and animation, as well as brightness and contrast adjustment, ZEN software (black edition, Zeiss, Germany) was used.

### Statistical Analyses

Data are expressed as mean ± SD unless otherwise stated. Groups were compared using two-tailed two-sample Student’s *t*-test or linear mixed model for multivariate data. All statistical analyses were completed using Microsoft Excel (Microsoft Corporation, Redmond, United States) or R and RStudio. Significance was determined as ^∗^*P* < 0.05, ^∗∗^*P* < 0.01, ^∗∗∗^*P* < 0.001.

## Data Availability Statement

All datasets generated for this study are included in the article/Supplementary Material.

## Ethics Statement

The animal study was reviewed and approved by Thüringer Landesamt für Verbraucherschutz.

## Author Contributions

DS and CE initiated, coordinated, and drove the project. DS organized and coordinated the mouse work and performed behavioral analysis including analysis of the X-ray recordings for diaphragm movement. DS and CH performed the characterization of Wt1+ cells (special and temporal distribution; marker analyses). CH and BP performed tissue clearing and light sheet microscopy. CH determined alterations in the neuronal subpopulations. DS, CH, BP, and CE wrote and revised the manuscript. All authors contributed to the article and approved the submitted version.

## Conflict of Interest

The authors declare that the research was conducted in the absence of any commercial or financial relationships that could be construed as a potential conflict of interest.
