# Supplementary figures and images for: Wt1 Positive dB4 Neurons in the Hindbrain Are Crucial for Respiration
Source: Front Neurosci. 2020 Nov 30;14:529487. doi: 10.3389/fnins.2020.529487 (PMC7734174; doi:10.3389/fnins.2020.529487)

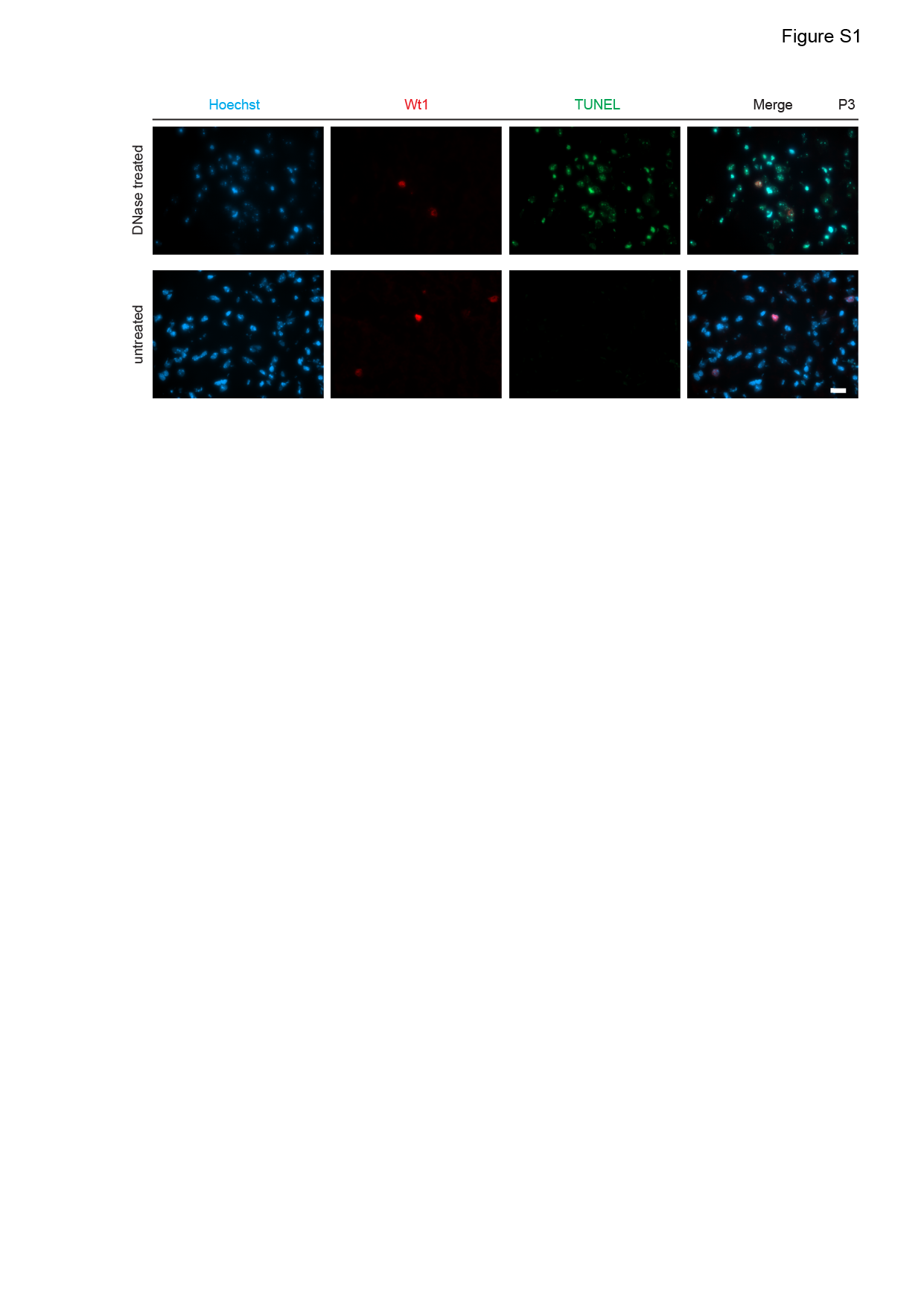

Supplement: Supplementary file 1 [file Image_1.TIF]
