# Supplementary material for: Wt1 Positive dB4 Neurons in the Hindbrain Are Crucial for Respiration
Source: Front Neurosci. 2020 Nov 30;14:529487. doi: 10.3389/fnins.2020.529487 (PMC7734174; doi:10.3389/fnins.2020.529487)
Supplement: Supplementary file 4 [file Table_1.DOCX]

**Suppl. Fig. 1** I**mmunoﬂuorescence staining of hindbrain sections from wild type newborns (P3).** TUNEL assay was performed to investigate for apoptotic Wt1+ cells. No apoptotic/TUNEL-positive Wt1+cells were observed. As positive control, sections were treated with DNase. Wt1 is depicted in red, TUNEL+ cells in green and Hoechst in blue. Scale bar: 20 μm

**Suppl. Vid. 1 3D- reconstruction of GFP labeled Wt1+ cells within the embryonic hindbrain.** The 3D reconstruction revealed one major and one minor column of Wt1+ cells per hemisphere in the ventral area of the medulla. The brain of a *Wt1^GFP^* reporter mouse was whole mount immunolabeled with an anti GFP antibody, cleared with *Scal*e and imaged via light sheet microscopy.

**Suppl. Vid. 2. Breathing of mice under high-speed X-ray recording.** X-ray fluoroscopy video shows skeleton and lung (bright grey area within mouse) of *Wt1^fl/fl^* control (with metal ring at the fur) and *Lbx1-ki-Cre;Wt1^fl/fl^* mouse (without further label) during respiration. The movement of the diaphragm was used to determine the respiration rate by counting number of inspiration over time (Screws are part of the cage).
